# Quantitative real-time PCR detection of Zika virus and evaluation with field-caught Mosquitoes

**DOI:** 10.1186/1743-422X-10-311

**Published:** 2013-10-22

**Authors:** Oumar Faye, Ousmane Faye, Diawo Diallo, Mawlouth Diallo, Manfred Weidmann, Amadou Alpha Sall

**Affiliations:** 1 Unité des Arbovirus et virus de fièvres hémorragiques, Institut Pasteur Dakar, 36, Avenue Pasteur, BP 220 Dakar, Senegal; 2 Unité d'entomologie médicale 36, Avenue Pasteur, BP 220 Dakar, Senegal; 3 Department of Virology, University Medical Center Göttingen, Göttingen, Germany

## Abstract

**Background:**

Zika virus (ZIKV), a mosquito borne flavivirus is a pathogen affecting humans in Asia and Africa. ZIKV infection diagnosis relies on serology–which is challenging due to cross-reactions with other flaviviruses and/or absence or low titer of IgM and IgG antibodies at early phase of infection- virus isolation, which is labor intensive, time consuming and requires appropriate containment. Therefore, real-time RT-PCR (rRT-PCR) is an appealing option as a rapid, sensitive and specific method for detection of ZIKV in the early stage of infection. So far, only one rRT-PCR assay has been described in the context of the outbreak in Micronesia in 2007. In this study, we described a one step rRT-PCR for ZIKV which can detect a wider genetic diversity of ZIKV isolates from Asia and Africa.

**Results:**

The NS5 protein coding regions of African ZIKV isolates were sequenced and aligned with representative flaviviruses sequences from GenBank to design primers and probe from conserved regions. The analytical sensitivity of the assay was evaluated to be 32 genome-equivalents and 0.05 plaque forming unit (pfu). The assay was shown to detect 37 ZIKV isolates covering a wide geographic in Africa and Asia over 36 years but none of the 31 other flaviviruses tested showing high analytical specificity. The rRT-PCR could be performed in less than 3 hours. This method was used successfully to detect ZIKV strains from field-caught mosquitoes.

**Conclusion:**

We have developed a rapid, sensitive and specific rRT – PCR for detection of ZIKV. This assay is a useful tool for detection of ZIKV infection in regions where a number of other clinically indistinguishable arboviruses like dengue or chikungunya co-circulate. Further studies are needed to validate this assay in clinical positive samples collected during acute ZIKV infection.

## Background

Zika virus (ZIKV) is an arbovirus *(Flaviviridae* family genus *flavivirus*[1] transmitted by mosquitoes*.* Its natural transmission cycle in Africa involves primarily *Aedes* species including *Ae. furcifer, Ae. taylori* and *Ae. luteocephalus* and *Cercopithecus aethiops*, *Erythrocebus patas* monkeys [2] while it is also transmitted by domestic mosquitoes *Aedes (Ae.) aegypti, Ae. hensilli*[3]. The survival of ZIKV in interepizootics is believed to depend on vertical transmission of the virus in *Aedes*[4].

Like all flaviviruses, ZIKV is a single-stranded RNA virus with a positive-polarity RNA genome of approximately 11 kb. Both termini of the genomic contain sequences that do not encode viral proteins, known as the 5′ and the 3′ untranslated region. The encoded polyprotein is translated and co- and posttranslationally processed by viral and cellular proteases into three structural (capsid [C], premembrane [prM] or membrane [M], and envelope [E]) and seven nonstructural (NS1, NS2a, NS2b, NS3, NS4a, NS4b, and NS5) proteins. The NS5 protein is constituted by two distinct domains, an N-terminal methyltransferase and a C-terminal RNA-dependent RNA polymerase that are required for capping and synthesis of the viral RNA genome, respectively [5-7].

ZIKV infection in humans symptoms ranges from asymptomatic to influenza like symptoms such as fever, headache, arthralgia, myalgia, malaise, anorexia, rash, asthenia, retro-orbital eye pain, oedema, lymphadenopathy, and diarrhoea [8-11]. The non-specific clinical presentation can be confused with most other arboviruses particularly dengue and chikungunya virus infection.

ZIKV, an emerging neglected virus, was first documented in 1947 when it was isolated from a sentinel rhesus monkey stationed on a tree platform in the Zika forest, Uganda [12]. Since then, epizootics and small epidemics have occurred in Africa and Asia [13]. In Africa, the first human isolated has been described during an outbreak of jaundice in eastern Nigeria by MacNamara, 1954 [13]. Then, serological and entomological Zika infection were reported in different area in Africa (Burkina Faso, Ivory Cost, Egypt, Central African Republic, Mozambic, Nigeria, Uganda, Central African Republic and Senegal) [2,9,14-19]. Between 1968 and 2002, 606 strains including 10 human ZIKV strains were isolated in Central and West Africa countries and reported by the WHO Collaborating Center for Arbovirus and Viral Hemorrhagic Fever of Pasteur Institute in Dakar [20]. In 2007, the outbreak in Yap (Micronesia) became the largest outbreak of ZIKV ever reported [21]. In Senegal, the entomological and virological surveillance program of arboviruses since 1972 showed an enzootic circulation of ZIKV [20]. In 2008, a probable non vector transmission of ZIKV were reported when two American scientists contracted the virus while working in Kedougou, South-Eastern Senegal. The transmission of ZIKV to the wife of one of the scientists who had no history travel in the virus endemic area supports sexual transmission of ZIKV in this case [22].

Currently diagnosis of ZIKV infection is based on detection of specific antibodies or virus isolation from animals or mosquitoes which are time consuming [13,23]. Standard RT-PCR and quantitative RT-PCR provide a rapid, specific and sensitive method for ZIKV early detection [21,22]. However, real-time PCR, in contrast to conventional assays, has many advantages, including rapidity, quantitative measurement, low contamination rate and easy standardization. To date, one rRT-PCR assay focusing on the detection of Micronesian ZIKV strains [21] is available but it does not cover the genetic diversity and geographic distribution of ZIKV. In this study, we developed a one step rRT-PCR assay capable to detect ZIKV strains circulating in Africa and Asia.

## Material and methods

### Viruses

Viral strains used were provided by WHO Collaborating Center for arboviruses and viral hemorrhagic fever (CRORA) at the Institut Pasteur de Dakar. ZIKV and other flaviviruses strains isolated from mosquitoes and non-human vertebrates used in this study are described in Tables 1 and 2. Viral stocks were prepared by inoculating viral strains into AP 61 monolayer continuous cell lines in Leibovitz 15 (L-15) growth medium (GibcoBRL, Grand Island, NY, USA) supplemented with 5% foetal bovine serum (FBS) (GibcoBRL, Grand Island, NY, USA), 10% tryptose phosphate, penicillin-streptomycin and fungizone (Sigma, Gmbh, Germany). After 7 days of propagation, viral infection was tested by an indirect immunofluorescence assay (IFA) using specific hyperimmune mouse ascitic fluids as previously described [23] and supernatants from infected cells were collected as stocks for virus RNA isolation. ZIKV stocks were used for sequencing and evaluation of the sensitivity of the rRT-PCR assay. Other flaviviruses were used to evaluate the specificity of the assay.

**Table 1:** Zika strains used in this study

**Reference**	**Hosts**	**Countries**	**Year of isolation**
ArD 7117	*Aedes luteocephalus*	Senegal	1968
ArD 9957	*Aedes furcifer*	Senegal	1969
ArD30101	*Aedes luteocephalus*	Senegal	1979
ArD 30156	*Aedes furcifer*	Senegal	1979
AnD 30332	*Cercopithecus aethiops*	Senegal	1979
HD 78788	Humain	Senegal	1991
ArD 127707	*Aedes furcifer*	Senegal	1997
ArD 127710	*Aedes taylori*	Senegal	1997
ArD 127984	*Aedes furcifer*	Senegal	1997
ArD 127987	*Aedes luteocephalus*	Senegal	1997
ArD 127988	*Aedes furcifer*	Senegal	1997
ArD 127994	*Aedes taylori*	Senegal	1997
ArD 128000	*Aedes luteocephalus*	Senegal	1997
ArD 132912	*Aedes dalzieli*	Senegal	1998
ArD 132915	*Aedes dalzieli*	Senegal	1998
ArD 141170	*Aedes dalzieli*	Senegal	2000
ArD 142623	*Anopheles coustani*	Senegal	2000
ArD 149917	*Aedes dalzieli*	Senegal	2001
ArD 149810	*Aedes dalzieli*	Senegal	2001
ArD 149938	*Aedes dalzieli*	Senegal	2001
ArD 157995	*Aedes dalzieli*	Senegal	2001
ArD 158084	*Aedes dalzieli*	Senegal	2001
ArD 165522	*Aedes vittatus*	Senegal	2002
ArD 165531	*Aedes dalzieli*	Senegal	2002
ArA 1465	*Aedes africanus*	Côte d’Ivoire	1980
ArA 27101	*Aedes opok*	Côte d’Ivoire	1990
ArA 27290	*Aedes opok*	Côte d’Ivoire	1990
ArA 27106	*Aedes luteocephalus*	Côte d’Ivoire	1990
ArA 27096	*Aedes africanus*	Côte d’Ivoire	1990
ArA 27407	*Aedes africanus*	Côte d’Ivoire	1990
ArA 27443	*Muci graham*	Côte d’Ivoire	1990
ArA 506/96	*Aedes vittatus*	Côte d’Ivoire	1996
ArA 975-99	*Aedes aegypti*	Côte d’Ivoire	1999
ArA 982-99	*Aedes vittatus*	Côte d’Ivoire	1999
ArA 986-99	*Aedes furcifer*	Côte d’Ivoire	1999
ArA 2718	*Aedes luteocephalus*	Burkina Faso	1981
ArB 1362	*Aedes africanus*	Republic Center Africa	1968
P6-740	*Aedes aegypti*	Malaysia	1966

**Table 2:** Flavivirus strains used in this study

**Flavivirus species**	**Reference**	**Hosts**	**Countries**	**Year of isolation**
Dengue 1	ArA 15120	*Aedes aegypti*	Côte d’Ivoire	1985
Dengue 2	ArD 63334	*Aedes furcifer*	Senegal	1989
	ArA 6894	*Aedes aegypti*	Burkina Faso	1986
	ArA 29982	*Aedes lutoecephalus*	Côte d’Ivoire	1992
	ArD 140 875	*Aedes furcifer*	Senegal	1999
	ArD 140 884	*Aedes lutoecephalus*	Senegal	1999
	ArD 141 069	*Aedes furcifer*	Senegal	1999
	ArD 141 070	*Aedes lutoecephalus*	Senegal	1999
	ArD 141073	*Aedes taylori*	Senegal	1999
	ArD 142 774	*Aedes fircifer*	Senegal	1999
Dengue 4	HD 38549	Human	Senegal	1983
Yellow Fever	ArA 408/78	Aedes luteocephalus	Côte d’Ivoire	1978
	HA 016/97	Human	Liberia	1997
	ArD 149213	*Aedes lutoecephalus*	Senegal	2000
	ArD 149 214	*Aedes furcifer*	Senegal	2000
West Nile	AF260968	Human	Egypt	1951
	M12294	Human	Uganda	1937
Usutu	ArD 130317	Culex perfuscus	Senegal	1998
Ss. Usutu	ArB 1803/69	*Culex perfuscus*	Central African Republic	1969
Bagaza	ArB 209	*Culex sp*	Central African Republic	1966
Bouboui	ArB 490	*Anopheles paludis*	Central African Republic	1967
Dakar Bat	AnD 249	*Scotiphilus sp*	Senegal	1962
Kedougou	ArD 14701	*Aedes minutus*	Senegal	1972
Koutango	AnD 5443	*Tatera kempi*	Senegal	1968
Ntaya	ArB 472	Culex sp	Central African Republic	1967
Uganda S	ArD 109325	Ades furcifer	Senegal	1994
Saboya	AnD 4600	*Tatera kempi*	Senegal	1968
Sepik	MK 7148	*Mansonia septempunctata*	New Guinea	1966
Spondweni	SA Ar 94	*Mansonia uniformis*	South African Republic	1955
Wesselsbron	ArB 4177	Rhipicephalus muhsamae a	Central African Republic	1982
Yaounde	ArY 276/68	*Culex nebulosus*	Cameroon	1968

### Titration of viral stocks

Viral stocks were titrated on Vero cells (African Green Monkey Kidney) supplemented with 10% fetal bovine serum (FBS) as well as penicillin-streptomycin (1%) and fungizone (0,05%). Viral suspension stock were serially 10-fold diluted in L-15 medium with 10% FBS. Two hundred microliters were inoculated on Vero cell monolayers in the wells of a 24-well plate. After 4 h of virus adsorption at 37°C, cells were overlaid with 3,2% of carboxymethylcellulose–L-15 medium containing 10% FBS. After incubation at 37°C for 7 days, cells were stained with 1% black amido, dried at room temperature and the plaques were counted.

### Primer and probe design

The NS5 sequences of ZIKV were chosen as target for the primers. ZIKV strains from Africa sequenced and desposited at Genbank (accession number, KF38304-KF383114), strain from Malaysia (NC_012532) and the strain related to Micronesian outbreak in 2007 (EU545988 ) were aligned using the Clustal W program [24]. A stretch of nucleotides conserved in the strains was identified and the primers and a short LNA probe sequence (16 nt) was designed using the Primers Express software and the LNA probe design window at http://lna-tm.com/[25]. The probe contained the fluorescent reporter dye 6-carboxyfluorescein (FAM) at the 5′-end and the fluorescent quencher dye 6-carboxytetramethylrhodamin (TAMRA) at the 3′-end. The primers and probe sequences and characteristics are shown in Table 3.

**Table 3:** Nucleotide sequences of primers and probe used in the qRT-PCR assay

	**Sequence 5′ – 3′**	**Nucleotide position**
Probe	FAM-CTYAGACCAGCTGAAR-BBQ	9304–9320
Forward primer	AARTACACATACCARAACAAAGTG GT	9271–9297
Reverse primer	TCCRCTCCCYCTYTGGTCTTG	9352–9373

FAM, 6-carboxyfluorescein; *BBQ*, Black Berry Quencher. Y = T or C, R = A or G.

### Generation of RNA standard for the rRT-PCR

An *in vitro* transcribed RNA of the NS5 gene of ZIKV strain ArD165531, was used to determine the detection limit of the assay. A size of 1083 bp of the NS5 region was amplified with the primers FD3/FU1 [1] and the product was purified using the QIAquick gel extraction kit (Qiagen GmbH, Heiden, Germany) and ligated to the pGEM-T vector (Promega, Madison, USA). The recombinant plasmid was used to transform *E. coli* X-Blue 1 strain (Invitrogen, Carlsbad, USA). The orientation of the insert DNA was confirmed by sequencing. The insert was amplified using the primers M13R/M13F and then purified using the QIAquick gel extraction kit. *In vitro* transcription and quantification of transcribed RNA was performed as previously described [26] with a slight modification consisting of DNA digestion using the DNAfree kit (Ambion, Austin, Texas, USA) instead of DNASE digestion and TRIZOL purification. The RNA quantification is performed with the fluorescence dye RiboGreen (Invitrogen, Eugene, Oregon, USA) which specifically binds to single strand RNA. The analysis is performed in a 96-well plate in the ABI-PRISM 7500 which is used as a fluorimeter. Quantification was performed in comparison to a RNA-standard of 20 ng/ml -1000 ng/ml RNA supplied with the kit and a standard curve was established. The copy of RNA (molecules/μl) was calculated as follows: C × A/L where C represents the concentration of RNA (g/mL) assessed by OD measurement, A is the Avogadro number (6.023 × 10^23^), and L is the length of the synthetic RNA (nucledotide) and 330 is an approximation of the molecular weight of a nucleotide (g/mol).

### Analytical sensitivity of the assay

The titer of ZIKV stocks were determined and 10-fold dilutions of the stock were used to study the sensitivity of the assay in a synthetic normal human plasma (Acrometrix, Benicia, CA), used in absence of naturally human infected ZIKV sample and Leibovitz 15 (L-15) growth medium (GibcoBRL, Grand Island, NY, USA), used as supernatants of infected AP61 cells with ZIKV.

### Viral RNA extraction

RNA was extracted from ZIKV stocks using the QIAamp RNA Viral Kit (Qiagen GmbH, Heiden, Germany) according to the manufacturer’s recommendations. Briefly, 100 ml of culture supernatants were mixed with AVL-Carrier RNA buffer. After 10 min incubation at room temperature, 400 ml of ethanol was added and samples were transferred into a column containing silica and centrifuged at 8000 g for 1 min. The RNA was washed twice with buffer AW1 and AW2, respectively. RNA was eluted in 50 μl of TE buffer and stored at –80°C until use.

### One-step real-time rRT-PCR

RNA was amplified by real-time RT-PCR in an ABI Prism 7500 SDS Real-Time cycler (Applied Biosystems, Foster City, USA). The Quantitect One-Step RT-PCR kit (Qiagen, Hilden, Germany) was used with a 25 μl reaction mixture under the following conditions: 0.25 μl of kit enzyme mixture (including reverse transcriptase RT and Taq polymerase), 10 μl of 2 × Quantitect RT-PCR buffer, 1.25 μl of 10 μM of each primer, 0.5 μl of 10 μM of probe at 10 μM, 6.8 μl of DNA RNA free water (Mol Bio grade, Hamburg, Germany) and 5 μl of the extracted sample. Each amplification run contained one negative and one positive control. The negative control consisted of blank reagent and water. For the positive control, nucleic acid extracted from virus stocks was used. The following thermal profile was used a single cycle of reverse transcription for 10 min at 50°C, 15 min at 95°C for reverse transcriptase inactivation and DNA polymerase activation followed by 40 amplification cycles of 15 sec at 95°C and 1 min 60°C (annealing-extension step). The data were analyzed using the SDS software from Applied Biosystems.

### Field-caught mosquitoes

Mosquitoes samples were collected during a routine surveillance for arbovirus in Kedougou, Southeastern Senegal from May to December 2011 as described previously [27] and pooled by species into groups of up 50 individuals. Monospecific mosquito pools were homogenized in 2.5 ml of L-15 medium containing 20% fetal bovine serum (FBS) and centrifuged for 20 min at 10,000 × g at 4°C. For the homogenate, 1 ml of the supernatant was inoculated into *Aedes pseudoscutellaris* as described previously [23]. Cells were incubated at 28°C. Within 10 days, slides were prepared for IFA against 7 pools of immune ascitic fluids specific for most of the African mosquito-borne arboviruses.

RNA was also extracted from supernatant of mosquitoes pools as described above and used for the ZIKV rRT-PCR assay. The mosquitoes pools were also screened for dengue an yellow fever using primers and probes described previously [28,29].

## Results

### Design and evaluation of primers and probes

**Figure 1:**
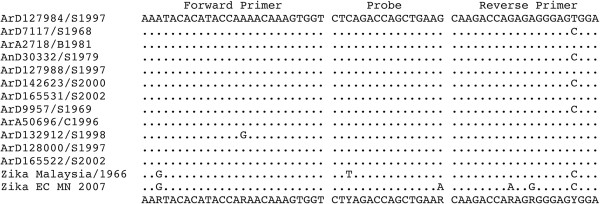
**Alignment of the designed primers and probe with Zika virus strain sequences.** Dots indicate identity with the consensus sequence on the top of the alignment.

An alignment of the NS5 sequences of 13 African and 1 Asian (Malaysia) ZIKV strains identified a highly conserved region of 102 nucleotides (nt) and highly divergent from other flaviviruses. A reverse primer (nt 9352 -TCCRCTCCCYCTYTGGTCTTG-9373), a forward primer (nt 9271- AARTACACATACCARAACAAAGTG GT-9297) and a 16 nt LNA-probe (nt 9304-FAM-CTYAGACCAGCTGAAR-BBQ -9320) were designed (Figure 1) (Table 3).

### Sensitivity and specificity of the rRT-PCR assay

**Figure 2:**
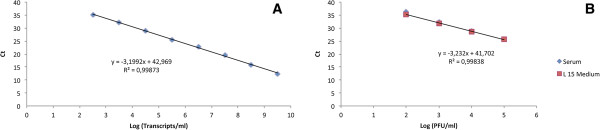
**Sensitivity of the rRT-PCR assay. (A)** Standard curve for Zika virus RNA transcript dilution series showing the threshold cycles Ct plotted against the log_10_ of Zika RNA transcripts and **(B)** Standard curve obtained with 10-fold serial dilutions of Zika virus. Ct values obtained are plotted against the log of the quantity of infectious virus (pfu/ml).

The assay detected the RNA of all 37 ZIKV strains (Table 1). The detection limit of the assay was evaluated using a quantitative RNA standard and a pfu-dilution series. To standardize the assay, serial dilutions of transcribed ZIKV RNA were tested by the real time PCR developed (Table 4). Three assays using transcript RNA prepared on different days were used to plot a standard curve (Figure 2A).

**Table 4:** Detection limit of the qRT-PCR assay for Zika virus

**Synthetic RNA /μl**	**nb positive/ nb tested**	**Ct**	**Percentage**
3,2 × 10^9^	3/3	12.15 ± 0.15	100
3,2 × 10^8^	3/3	15.76 ± 0.21	100
3,2 × 10^7^	3/3	19.32 ± 0.25	100
3,2 × 10^6^	3/3	22.62 ± 0.27	100
3,2 × 10^5^	3/3	25.28 ± 0.31	100
3,2 × 10^4^	3/3	28.50 ± 0.46	100
3,2 × 10^3^	3/3	31.77 ± 0.39	100
3,2 × 10^2^	3/3	34.79 ± 0.43	100
3,2 × 10^1^	0/3	neg	0
3,2 × 10^0^	0/3	neg	0

The detection was linear over 7 log_10_ steps from 3,2 × 10^9^ (Ct 12.15) to 3,2 × 10^2^ molecules detected (Ct 34.79) and the regression coefficient (R^2^ = 0.9987) indicates that the assay is highly reproducible (Figure 2A). Intra-assay and interassay coefficient variation (CV) of the mean Ct values ranged from 1.19–1.61% and 1.23–2.72% respectively when using synthetic RNA. The lowest detection limit of the rRT-PCR assay was also evaluated using ten-fold dilutions of a ZIKV stocks ranging from 5 × 10^4^ to 5 × 10^-6^ PFU/ml in L15 medium or synthetic normal human plasma. The result showed that the detection limit of the established rRT-PCR assay was 0.05 pfu / reaction in L15 medium and normal human plasma. The Ct values obtained were highly correlated (R^2^ = 0.9984) and ranged from 25.60 to 36.24 (Table 5, Figure 2B). The Cts across the quantitative range showed a standard deviation ranging from 1.13 to 1.61 Ct. The RNA of 37 ZIKV strains and 31 flavivirus strains (Table 2), was tested and no cross detection was observed indicating a high specificity of the assay.

**Table 5:** Sensitivity of the qRT-PCR assay for Zika virus detection

**PFU/ml**	**Ct**	
	**serum**	**L15 medium**
50 000	25.60 ± 1.138	25.70 ± 1.138
5000	28.88 ± 1.443	28.58 ± 1.443
500	32.23 ± 1.604	31.92 ± 1.604
50	36.24 ± 1.61	35.36 ± 1.61
5	ND	ND
0.5	ND	ND

### Evaluation of the real-time RT-PCR assay analyzing mosquito and serum samples

Overall a total of 1969 pools of mosquitoes collected at different sites in Kedougou between May to December 2011 were tested using the ZIKV rRT-PCR assay. ZIKV was detected in 31 mosquito pools out of 1969 from Aedes and Mansonia genus. A total of 15 out of 31 (49%) mosquito pools were found positive by rRT-PCR, while 7 (22%) mosquitoes pools were positive by virus isolation and 9 (29%) were positive by both tests, with Ct values ranged between 20 to 35. The rRT-PCR method was significantly more sensitive than virus isolation (Khi2 test, p = 0.0371). No mosquito pool was found positive for dengue and yellow fever virus. *Aedes africanus*, *Aedes furcifer* and *Aedes luteocephalus* species were the most infected vectors with 16% positive (5/31 strains) each. The result showed that 87% (27/31 strains) of the positive pools were collected in June and September to December. NS5 region of positive mosquito pools for ZIKV NS5 were amplified and sequenced using primers FD3/FU1 described previously [1] A blast alignment of the ZIKV NS5 sequences showed 97 to 100% similarity with ZIKV strain ArD41519 (accession number HQ234501) isolated in Kedougou, South-Eastern Senegal, in 1984.

## Discussion

In West Africa, ZIKV epizootics are regularly detected [20] but few human cases are reported. Underreporting might be due to the circulation of other arboviruses which cause similar clinical features as ZIKV infection. Detection of ZIKV so far is based and virological methods, which are time consuming. A previously developed RT-PCR was adapted for real time PCR detection of ZIKV in samples from human cases during the 2007 Micronesian outbreak [21].

In this study, we have developed a new sensitive and specific one step rRT-PCR for detection of ZIKV in serum and cell culture supernatants using a Taqman probe containing locked nucleotides, in the NS5 region of ZIKV genome using sequences of ZIKV strains circulating in Africa and Asia. Indeed, isolates from Malaysia and the outbreak of Micronesia are closely related [30].

Real-time RT-PCR for the diagnosis of acute ZIKV infection has many advantages compared to virus isolation and conventional RT-PCR. Virus isolation is considered as the “gold standard” for diagnosis of viral infection [31]. However, it has the disadvantage of low sensitivity and needs more than 10 days. Compared to conventional RT-PCR, real-time RT-PCR has several advantages such as rapidity, low risk of false positive results, high sensitivity, specificity and the possibility of quantitative measurements.

The assay was tested on ZIKV isolates from various geographical locations (Senegal, Cote d’ivoire, Burkina Faso and Central African Republic), hosts (mosquito, human and monkey) covering a period of 36 years. Compared to the only available assay based on ZIKV samples obtained during the outbreak in Micronesia, the advantage of our assay is that the primers and probe of our rRT-PCR assay were designed and evaluated using genetic diversity and geographic distribution of ZIKV isolated over 36 years. The developed rRT-PCR assay allowed the detection of 0.5 pfu/ml. This detection limit value is similar to that found by Wu *et al.,*[29] and is therefore sensitive enough to diagnose ZIKV in clinical cases. Indeed, viraemia found in human infection ranges from 10^2^ to 10^6^ pfu/ml [8,10,21,32]. The limit of detection by testing 10 fold-dilutions normal human plasma and L-15 growth medium for AP61 cell was the same. ZIKV was detected up to dilution 50 pfu/ml both in normal human plasma. and L-15 medium. This finding suggests a high reproducibility of the ZIKV rRT-PCR assay. Moreover, the rRT-PCR assay established for ZIKV was found to be more sensitive than that of the traditional RT-PCR assay developed previously [33]. This result confirms the greater sensitivity of real-time PCR compared to conventional RT-PCR [34]. The reproducibility of the real time PCR assay was high as shown by intra- and inter-assay variation analysis. The assay may improve early identification of acute Zika fever, and implementation of early treatment and control measures during ZIKV outbreaks. However, in this study, our assay was evaluated using synthetic normal human plasma. Then, further studies using humans sera naturally infected by ZIKV are needed to validate this assay.

For a better assessement of the established rRT-PCR, the specifity was tested with a variety of mosquito-borne and vertebrate-borne flaviviruses as described in Materials and methods. No cross reaction was observed demonstrating the high specificity this assay. The specificity of the rRT-PCR assay allows its use for differential diagnosis of arboviral infections in Africa and Asia where ZIKV co-circulates with other arboviruses such as Dengue, Chikungunya and yellow fever viruses [22].

Virus isolation has documented a permanent circulation of ZIKV in Southeastern Senegal since 1972 [2,13]. The TaqMan assay was able to detect ZIKV in field-collected mosquito pools at higher sensitivity than virus isolatiob in AP61 cells (Khi2 test, p = 0.037). However, 7 positive mosquitoes pool was found only by virus isolation and negative by rRT-PCR assay.

This might be explained by the loss of the ZIKV RNA during the extraction process or during freezing and thawing the samples prior to the rRT-PCR test. Furthermore, no mosquito pool was found positive for dengue and yellow fever, major abovirus which co-circulate with ZIKV in Kedougou aera. In addition, ZIKV NS5 region of the positive mosquito pools showed 97 to 100% identity with senegalese ZIKV strain ArD41519 (accession number HQ234501), confirming the specificity of the assay. It will therefore help to improve screening for ZIKV in mosquito pools.

Like others arbovirus (YFV, DENV, CHIKV) in this Kedougou region, ZIKV were found frequently at the end of the rainy season between September and December [2,35], characterized by the existence of a very diversified mosquito fauna and an old population of vectors that have taken several blood meals and thus are more likely to be infected by contact with viremic hosts. ZIKV was also detected in mosquito vectors as collected in June, corresponding to the beginning of the rainy season and the mosquito activities in southern Senegal. These detections suggest a rapid and huge amplification of the virus in the vectors that might be infected through vertical transmission and/ or from vertebrate reservoirs [4].

## Conclusion

The expansion of ZIKV outside Africa shows the need to develop rapid assays and specific monitoring of the virus. Rapid detection of the virus in field-collected specimens can accelerate appropriate mosquito control measures that could prevent transmission and disease among human population. In this study we developed a rapid, sensitive and specific real time PCR for the detection of ZIKV circulating in Africa and Asia. This assay will be a useful tool for differential Zika fever diagnostics in a situation where number of other disease like malaria, dengue, chikungunya co-circulate and are clinically indistinguishable.

## Competing interests

The authors declare that they have no competing interests.

## Authors’ contributions

Oum F, Ous F, MD, MW and AAS designed the study, analyzed the data and wrote the manuscript. Oum F and DD performed the experiment. All the authors have read and approved the final manuscript.
